# Free-Living Turtles Are a Reservoir for Salmonella but Not for *Campylobacter*

**DOI:** 10.1371/journal.pone.0072350

**Published:** 2013-08-07

**Authors:** Clara Marin, Sofia Ingresa-Capaccioni, Sara González-Bodi, Francisco Marco-Jiménez, Santiago Vega

**Affiliations:** 1 Instituto de Ciencias Biomédicas. Departamento de Producción Animal, Sanidad Animal, Salud Pública Veterinaria y Ciência y Tecnología de los Alimentos, Facultad de Veterinaria, Universidad CEU-Cardenal Herrera, Alfara del Patriarca, Valencia, Spain; 2 Instituto de Ciência y Tecnología Animal, Universidad Politécnica de Valencia, Valencia, Spain; Iowa State University, United States of America

## Abstract

Different studies have reported the prevalence of *Salmonella* in turtles and its role in reptile-associated salmonellosis in humans, but there is a lack of scientific literature related with the epidemiology of *Campylobacter* in turtles. The aim of this study was to evaluate the prevalence of *Campylobacter* and *Salmonella* in free-living native (*Emys orbicularis*, n=83) and exotic ( *Trachemys* *scripta* *elegans*, n=117) turtles from 11 natural ponds in Eastern Spain. In addition, different types of samples (cloacal swabs, intestinal content and water from Turtle containers) were compared. Regardless of the turtle species, natural ponds where individuals were captured and the type of sample taken, *Campylobacter* was not detected. *Salmonella* was isolated in similar proportions in native (8.0±3.1%) and exotic (15.0±3.3%) turtles (p=0.189). The prevalence of *Salmonella* positive turtles was associated with the natural ponds where animals were captured. Captured turtles from 8 of the 11 natural ponds were positive, ranged between 3.0±3.1% and 60.0±11.0%. Serotyping revealed 8 different serovars among four *Salmonella enterica* subspecies: *S. enterica* subsp. *enterica* (n = 21), *S. enterica* subsp. *salamae* (n = 2), *S. enterica* subsp. *diarizonae* (n = 3), and *S. enterica* subsp. *houtenae* (n = 1). Two serovars were predominant: S. Thompson (n=16) and *S* *. typhimurium* (n=3). In addition, there was an effect of sample type on *Salmonella* detection. The highest isolation of *Salmonella* was obtained from intestinal content samples (12.0±3.0%), while lower percentages were found for water from the containers and cloacal swabs (8.0±2.5% and 3.0±1.5%, respectively). Our results imply that free-living turtles are a risk factor for *Salmonella* transmission, but do not seem to be a reservoir for *Campylobacter* . We therefore rule out turtles as a risk factor for human campylobacteriosis. Nevertheless, further studies should be undertaken in other countries to confirm these results.

## Introduction

Campylobacteriosis and salmonellosis are the two most prevalent zoonoses worldwide [1]. These zoonoses represent an important public health problem and controlling the disease has become a vital challenge in most countries [1–7]. Campylobacteriosis and salmonellosis were responsible respectively for 212,064 and 99,020 cases of illnesses in the EU [1]. Moreover, campylobacteriosis is the most common cause of acute bacterial gastroenteritis in the EU [1,8,9].

Apart from acute gastroenteritis, campylobacteriosis may lead to more severe, occasionally long-term sequelae such as Guillain–Barré syndrome, reactive arthritis and irritable bowel syndrome [10,11]. The high and rapidly increasing incidence and the capacity of *Campylobacter* to cause considerable morbidity make campylobacteriosis a public health problem of considerable magnitude [2]. However, compared to *Salmonella* , few outbreaks are reported, and most cases of campylobacteriosis are considered to be “sporadic” rather than a part of recognised outbreaks, with a seasonal peak during summer [12].

*Campylobacter* are commensally widespread in the intestines of wild and domesticated animals, resulting in contamination of the environment, including water sources [13]. Although *Campylobacter* is mostly perceived as a food-borne pathogen, there is evidence for other transmission pathways, including direct and indirect contact with infected animals, people and environment [2,14–17]. In recent years, the popularity and number of exotic reptiles kept as pets has risen, leading to an increase in the number of reptile-associated zoonotic pathogens infections, especially in vulnerable patients such as infants, young children, the elderly or immunocompromised adults [18–23]. In this way, turtles represent a special risk, as they are commonly kept as pets for children [24]. Similar to *Campylobacter* , *Salmonella* infections are caused by consumption of contaminated food, person-to-person transmission, waterborne transmission and numerous environmental and animal exposures [25]. Specifically, reptiles and other coldblooded animals (often referred to as ‘exotic pets’) can act as reservoirs of *Salmonella* , and cases of infection have been associated with direct or indirect contact with these animals [3]. *Trachemys* *scripta* *elegans* is the most common pet turtle worldwide and has been identified as an important source of infection in human cases and outbreaks of salmonellosis since 1963 [26–37]. For this reason, the epidemiology of pathogenic microorganisms in free-living and pet turtles has been studied [7,24,38–40]. In particular, results from these studies have shown that the incidence of *Salmonella* in pet turtles ranged from 0% to 72.2% [5,39,41,42] and from 0% to 15.4% in free-living turtles [39,43–47]. To our knowledge, no prevalence studies of *Campylobacter* in pet and free-living turtles have been carried out.

In this context the aim of this study was to assess the prevalence of *Campylobacter* and *Salmonella* in free-living native (*Emys orbicularis*) and exotic turtles ( *Trachemys* *scripta* *elegans*) located in 11 natural pond areas in Eastern Spain (Valencia Region). Additionally, we assessed the relative sensitivity of different sample types (cloacal swabs, intestinal content and water from containers) to estimate *Salmonella* prevalence in turtles.

## Material and Methods

The Ethics and Animal Welfare Committee of the Universidad CEU Cardenal Herrera approved this study. All animals were handled according to the principles of animal care published by Spanish Royal Decree 1201/2005 (BOE, 2005; BOE = Official Spanish State Gazette). The Conselleria de Infraestructuras, Territorio y Medio ambiente (regional administration) gave permission to take samples. This project is included in the LIFE + Biodiversity section, which aims to develop innovative projects or demonstrations that contribute to the implementation of the objectives of the Commission communication (COM (2006) 216 final) "Halting the loss of Biodiversity for 2010- and beyond.”

During the period between July and October 2012, 200 free-living turtles were captured from 11 natural ponds in Eastern Spain (Pego-Oliva, Almenara, Castellón, Xeraco, Peñíscola, Villanueva de Alcolea, Pobla Tornesa, Cabanes, Vaca River, Moros and La Safor). After capture, each individual was housed singly in a plastic container with 2 litres of sterile water to prevent bacterial transmission among them. As bacteria excretion is not continuous, water samples were taken after two days in captivity. This study was undertaken within the framework of an eradication programme for exotic turtles. Therefore, native turtles were returned to their habitat after sampling, while exotic turtles were euthanised by sodium pentobarbital injection before taking the samples (Dolethal, Vétoquinol, E.V.S.A).

For each individual, two cloacal samples were taken using sterile cotton swabs (Cary Blair sterile transport swabs, DELTALAB^®^). After 2 days in the container, two water samples from plastic containers were collected. Each sample was analysed for *Campylobacter* and for *Salmonella* isolation. For exotic turtles, after euthanisation 2 cm of large intestine were collected and the content was homogenised.

### Detection of *Campylobacter* spp

The procedure was based on ISO 10272:2006 recommendations (Annex E). Intestinal content and swabs were directly streaked onto the two selective agar plates (mCCDA and Preston, AES laboratories®, Bruz Cedex, France) and incubated at 41.5±1^°^C for 44±4 hours. Water samples were pre-enriched in 1: 10 vol/vol Bolton Broth (OXOID, Dardilly, France) and then pre-incubated at 37±1^°^C for 5±1hours. Afterwards, 100 µl of the sample was cultured on the two selective agar plates as described above. All plates and broths were incubated in a micro-aerobic atmosphere (84% N_2_, 10% CO_2_ and 6% O_2_) generated in a gas charged incubator (CampyGen, Oxoid). Plates were examined for grey, flat, irregular and spreading colonies typical of *Campylobacter* . One putative colony was subcultured from each plate onto sheep blood agar for confirmation as *Campylobacter* spp. *Campylobacter* confirmation was performed by a mobility test using a dark field microscope, by oxidase and catalase biochemical test and by streaking at different temperatures and atmospheres on Columbia blood agar (AES Laboratories ®, Bruz Cedex, France). Finally, characterisation of the bacteria species was done with a hippurate hydrolysis test.

### Detection of *Salmonella* spp

The procedure was based on ISO 6579: 2002 recommendations (Annex D). Samples were pre-enriched in 1: 10 vol/vol Buffered Peptone Water 2.5% (BPW, Scharlau®, Barcelona, Spain) and then incubated at 37±1^°^C for 18±2 hours. The pre-enriched samples were transferred onto Semi-Solid Modification Rappaport Vassiliadis agar plate (MSRV, Difco®, Valencia, Spain) and incubated at 41.5±1^°^C for 24-48 hours. The culture obtained in MSRV was inoculated onto Xylose-Lysine-Desoxycholate (XLD, Liofilchem®, Valencia, Spain) and Xylose-Lysine-Tergitol-4 (XLT4, Biokar Diagnostics®, Pantin Cedex, France) and incubated at 37±1^°^C for 24-48 hours. After incubation, 5 typical colonies were streaked onto the surface of pre-dried nutrient agar plates (Scharlab®, Barcelona, Spain) 37±1^°^C for 24±3 hours. Then, a biochemical test using API (API-20®, bioMérieux, Madrid, Spain) was performed to confirm *Salmonella* spp. Moreover, *Salmonella* strains isolated were serotyped by the Ministry of Agriculture, Fisheries and Food Reference Laboratory (Algete, Madrid, Spain) in accordance with Kauffman-White-Le-Minor technique.

### Statistical analyses

A generalised linear model, which assumed a binomial distribution for *Salmonella* shedding, was fitted to the data to determine whether there was an association with turtle species (native and exotic), natural ponds where turtles were captured (Pego-Oliva, Almenara, Castellón, Xeraco, Peñíscola, Villanueva de Alcolea, Pobla Tornesa, Cabanes, Vaca river, Moros and La Safor) and sample type (cloacal swabs, intestinal content and water from the containers). A P value of less than 0.05 was considered to indicate a statistically significant difference. Data are presented as least squares means ± standard error of the least squares means. All statistical analyses were carried out using a commercially available software program (SPSS 16.0 software package; SPSS Inc., Chicago, Illinois, USA, 2002).

## Results

For *Campylobacter* isolation, overall 517 samples were analysed; 200 samples were from water containers (117 from exotic and 83 from native turtles), 200 from cloacal swabs (117 from exotic and 83 from native turtles) and 117 from intestinal content (only from exotic turtles). Regardless of the turtle species captured, the natural pond where animals were captured and the sample type (water from the container, cloacal swabs and intestinal content), *Campylobacter* was not detected.

For *Salmonella* isolation, overall 517 samples were examined; 200 samples were from water from the container (117 from exotic and 83 from native turtles), 200 from cloacal swab (117 from exotic and 83 from native turtles) and 117 from intestinal content (only from exotic turtles). Independently of the species of turtle analysed, 11.0±2.3% of the turtles tested positive. Moreover, of the exotic turtles sampled and the native turtles sampled, 15.0±3.3% and 8.0±3.1% were positive, respectively. No significant differences were found between the percentage of *Salmonella* and the turtle species studied. *Salmonella* was detected in exotic and native turtles from eight natural ponds investigated (Table 1). In positive natural ponds significant differences were found. The mean prevalence of *Salmonella* was 16.2±4.6% (ranged between 3.0±3.1% to 60.0±11.0%). In the natural ponds of Cabanes, Pobla Tornesa and Xeraco, *Salmonella* was not isolated.

**Table 1:** Percentage of *Salmonella* -positive turtles from different natural ponds.

**Natural pond**	***n***	** *Salmonella* (%)**
Peñíscola	20	10.0±6.7^a^
Moro	16	6.0±6.1^a^
Vaca river	22	5.0 ±4.4^a^
La Safer	24	8.0 ±5.6^a^
Almenara	20	60.0±11.0^b^
Pego-Oliva	22	3.0 ±3.1^a^
Castellón	17	24.0±10.3^a^
Villanueva de Alcolea	7	14.0 ±13.2^a^

*n:* number of samples tested. a, b: Different superscripts represent significant differences(P≤0.05). Data are presented as least squares means ± standard error of the least squares means.

The serovars isolated in native free-living turtles did not coincide with those isolated in exotic free-living turtles, except for S. Thompson. Serotyping revealed 8 different serovars among four *Salmonella enterica* subspecies (Table 2); *S. enterica* subsp. *enterica* (n = 21, 77.7%), *S. enterica* subsp. *salamae* (n = 2, 7.4%), *S. enterica* subsp. *diarizonae* (n = 3, 11.1%), and *S. enterica* subsp. *houtenae* (n = 1, 3.7%). No more than one serovar was isolated per individual. The most prevalent serovars isolated were S. Thompson (n=16, 59.2%) and *S* *. Typhimurium* (n=3, 11.1%).

**Table 2:** *Salmonella* serovars isolated from native and exotic free-living turtles.

**Turtle specie**	**n**	**Subspecies**	**Serovar**
Native *Emys orbicularis*	2	*enterica*	Thompson
	2	*enterica*	Baildon
	1	*salamae*	4,12:b[-]
	1	*salamae*	17:b[e,n,x,z]
	2	*diarizonae*	16:1,v[1,5,7]
Exotic *Trachemys* *scripta* *elegans*	3	*enterica*	Typhimurium
	14	*enterica*	Thompson
	1	*houtenae*	44:z4,z23: -
	1	*diarizonae*	38:1,v:z35

n: number of strains isolated

Significant differences for *Salmonella* detection were found among the different type of samples collected (Table 3). The highest isolation of *Salmonella* was obtained from intestinal content samples (12.0±3.0%), while for water from the containers and cloacal swabs lower percentages were found (8.0±2.5% and 3.0±1.5%, respectively).

**Table 3:** Percentages of *Salmonella* spp. detection for the different types of samples analysed.

**Sample type**	**N**	** *Salmonella* (%)**
Intestine	117	12.0±3.0^a^
Water from container	200	8.0±2.5^ab^
Cloacal swabs	200	3.0±1.5^b^

*n:* number of samples tested. a, b: Different superscripts represent significant differences(P≤0.05). Data are presented as least squares means ± standard error of the least squares means.

## Discussion

Symptoms of *Campylobacter* (fever, abdominal cramps and diarrhoea) are clinically indistinguishable from those of bacterial gastroenteritis caused by other organisms, such as *Salmonella* or *Shigella* species [48,49]. Pet turtles are considered an important reservoir for *Salmonella* [39,46,50] and we tested the hypothesis that free-living turtles would also be an important reservoir for *Campylobacter* . Members of the *Campylobacter* genus naturally colonise humans, farm animals, wild mammals, birds, reptiles and shellfish [51]. However, to date only two reports identify *Campylobacter* *foetus* of turtle origin as a human pathogen [38,52]. In the present study, *Campylobacter* was not detected. As for *Salmonella* isolation*,* *Campylobacter* detection is likely to be highly dependent on the choice of an adequate sampling procedure combined with a sensitive culture method [53,54]. However, direct plating of faecal samples has been shown to yield the best isolation efficiency for detection of *Campylobacter* [54,55]. One possible explanation for the lack of detectable *Campylobacter* from cloacal swabs is a lack of appreciable faecal material. Nevertheless, in our study neither cloacal swabs nor intestinal content were positive. Although molecular methods (PCR and qPCR) have several advantages over classical bacteriology for *Campylobacter* detection, a high level of agreement between both methods has been reported, especially with faecal samples [54,56]. Nevertheless, if *Campylobacter* had been present, it seems highly unlikely that the bacteria would not have been isolated in any of the samples analysed. Thus, our results show that free-living turtles appear not to be a reservoir for *Campylobacter* and we therefore rule out turtles as a risk factor for human campylobacteriosis.

The prevalence of *Salmonella* detected in this study among free-living Valencian turtles was moderate (11.0±2.3%) and consistent with those of other studies [39,47,57]. To our best knowledge, few studies on the prevalence of *Salmonella* in free-living turtles have been carried out in Spain [39,46,50]. However, contradictory results are present in the literature, since some authors revealed low prevalence of *Salmonella* in free-living turtles [39,43,45,57–59], while other authors reported a medium and high prevalence [42,50,60,61]. Free-living turtles are believed to shed *Salmonella* at lower rates than captive turtles because they are less or not even exposed to stress factors that increase shedding rates, or because they are not natural carriers of the bacteria [45,58,62]. However, for other authors free-living turtles are considered an important reservoir for *Salmonella* [46].

The serovar most frequently identified was S. Thompson, isolated in both exotic and native turtles. S. Baildon 9,46: a: [e, n, x], S. 4,12: b: [-], S. 17: b: [e, n, x] and S. 16,1, v [1,5,7] were also isolated in native turtles whereas *S* *. Typhimurium* , *S.* 44: z4,x23: [-] and S. 38:1, v: z35 were identified in the exotic ones. All serovars identified have previously been reported in reptiles and have been associated with human salmonellosis [63–68]. Although many of these serovars may be considered as types rarely associated with human disease, 10% or more of isolates belong to subsp. *enterica*, which comprises potential human pathogens, e.g. *S* *. Typhimurium* . This is, together with *S* *. Enteritidis* , one of the most frequently reported serovar involved in human salmonellosis [1,35]. The infections in reptiles are usually asymptomatic, although clinical salmonellosis in reptiles has been reported with the following symptoms: septicaemia, salpingitis, dermatitis, osteomyelitis and granulomatous disease [69]. In addition, *Salmonella* subsp. *houtenae* has been recently associated as a cause of meningitis in a child [63].

In the cloaca of turtles, the presence of *Salmonella* was lower than in the intestinal content. The lower recovery of *Salmonella* from cloacal swabs was probably due to wild turtles shedding *Salmonella* at lower rates because they are less stressed, as mentioned above [45,58,62]. Cloacal swabs appeared to be less sensitive than faecal samples [70]. As *Salmonella* excretion is not continuous [39], in our study turtles were kept for two days in water containers to increase shedding rates. As expected, analyses also indicated that stress increased shedding of *Salmonella* . Specifically, keeping the turtles for 48 hours in containers could increase the sensitivity of water samples, as suggested by our findings. For this reason, this sampling method may be applied in further studies to determine the prevalence of *Salmonella* in turtles.

This study showed free-living turtles as a risk factor for *Salmonella* infection, but our findings also indicate that free-living turtles appear not to be a reservoir for *Campylobacter* and we therefore discard the turtles as a risk factor for human campylobacteriosis. To our best knowledge, this is the first study in which campylobacteriosis is investigated in relation to free-living turtles as a possible reservoir. Nevertheless, further studies should be undertaken in other countries to confirm these results.
